# Experiences of women, men and healthcare workers accessing family planning services in Malawi: A grounded theory

**DOI:** 10.4102/safp.v62i1.5153

**Published:** 2020-10-07

**Authors:** Idesi T. Chilinda, Alison Cooke, Dame T. Lavender

**Affiliations:** 1 Department of Community Health, University of Malawi, Lilongwe, Malawi; 2 Division of Nursing, Midwifery and Social Work, University of Manchester, Manchester, United Kingdom

**Keywords:** family planning, contraception, unmet needs, Malawi, grounded theory, experiences, women, healthcare workers, men

## Abstract

**Background:**

The importance of modern contraceptive methods in averting unwanted pregnancies has been acknowledged in Malawi. Currently, the country has registered the highest rates of unsafe abortions, unmet needs for contraception and a low contraceptive prevalence rate. Understanding why these rates exist is important. However, women’s views and experiences regarding uptake of family planning methods in Malawi have not been explored.

**Methods:**

A grounded theory methodology was used. Data were gathered through in-depth interviews with women (*n* = 18), men (*n* = 10), healthcare workers (*n* = 10) and non-participant observations of family planning clinic consultations (*n* = 10). Data were analysed using constant comparative technique. Methods of open, axial and selective coding enabled subsequent conceptualisations until theoretical saturation occurred.

**Results:**

The core category ‘disenabling environment prevents women’s family planning needs from being met’ provides an understanding of women’s, men’s and healthcare workers’ experiences of contraceptive use and non-use. The disenabling environment contributed to shaping women’s family planning experiences. This was supported by three main categories: navigating the processes, disempowerment of women and learning by chance.

**Conclusion:**

Findings from this study illuminate contextual issues into how women, men and healthcare workers experience family planning use and non-use in Malawi. A multifaceted strategy is required to support a woman’s family planning needs. At community level, awareness and education of family planning methods is required to actively inform all people in society so that they support a woman’s family planning needs. At national level, laws that would empower women with decision-making ought to be developed and enforced.

## Introduction

Contraceptive use in low-resource countries, including sub-Saharan Africa, remains low, with a minimal rise from 23.6% to 28.5% between 2008 and 2015.^1^ There are rising rates of unmet need for contraception, which have caused concern to the global health community.^2^ Unmet need for contraception is when a woman is fecund and desires to use contraception but does not have access to it.^3^ Recent evidence indicates that reducing unmet needs for contraception remains the greatest challenge globally because of continuing unavailability of the service.^4^ It has been estimated that married women between the ages of 15–49 years in Malawi have an unmet need for family planning of 59%.^5^ This is despite the service being provided at no cost in all public health facilities. The unmet need for family planning in Malawi is associated with negative health outcomes. This is evidenced by a high rate of unintended pregnancies (currently at 54.2%) and a contraceptive prevalence rate of 56%.^6^ Recently, the number of women having induced, unsafe abortions in Malawi has risen to 70 000 every year.^7^ Consequently, the country is lagging in terms of achieving the Sustainable Development Goals (SDGs) and the post-Millennium Development Goals (MDGs) with a maternal mortality rate of 439/100 000 live births.^7^ Prior studies conducted in Malawi reported that fears and misconceptions of contraceptive use, lack of awareness and spousal disapproval of contraception limit widespread use of contraception whilst family planning providers lack necessary equipment to provide long-acting intrauterine contraceptive devices.^8,9,10,11^ Therefore, increasing family planning usage remains an unfinished health agenda for the government. Nevertheless, there is a dearth of evidence to explore the factors that hinder or facilitate women to use family planning methods in a Malawian context. This study sought to understand the experiences of women, men and healthcare workers regarding use and non-use of family planning methods at three health centre settings in Malawi. Gaining an in-depth understanding of the reasons why women are not using contraception is pivotal in developing a theory that would inform future family planning strategies.

## Methods

### Research design

Grounded theory methodology, by Strauss and Corbin,^12^ derived from pragmatism and symbolic interactionism^13^ was employed to achieve the aims of this study. This methodology aims to generate theories for areas where there is a paucity of evidence or if a new perspective is required. Because of this, it adopts an iterative and inductive process that allows the theory to emerge from participant’s accounts.^13^ The use of foreknowledge, theoretical sensitivity, field notes, memos and technical literature added strength to the research design.^12^ This enabled the experiences of women, men and healthcare workers to be contextually grounded.

### Study setting

The study was conducted at Lumbadzi, Area 25 and Area 18 health centres in Lilongwe district of Malawi. The health centres are owned by the government. Their physical infrastructure was noted to be similar. The similar set-up of the health centres allowed the researcher to obtain rich data that presented a broader view of family planning experiences in a Malawian context. The similar set-up of health centres would make findings of this study to be transferable to a wider population in other similar settings. The health centres were selected because they offer accessible free family planning services to the public and serve a high catchment population. Moreover, these health centres were conveniently selected because of their geographical accessibility to the researcher. All three health centres were providing both long-term and short-term family planning services to women. This allowed the women to have a wider choice of contraception.

Family planning methods such as implants, male and female condoms, pills and injectable Depo-Provera are available in all the health centres. Permanent methods of contraception (male and female sterilisation) are provided by Banja la Mtsogolo (a non-governmental organisation of Marie Stops International that provides contraception and safe abortion to women in developing countries). Participants from this study were accessed from two urban and one semiurban community settings within the catchment areas of the health centres.

### Participants

In keeping with the Straussian approach, an initial sample of three participant groups was included to participate in the study. The inclusion criteria were: (1) women between the ages of 18–45 years who had an unmet need for contraception, (2) healthcare workers who were providing family planning services and (3) men who were not using any contraception and willing to provide their perspectives. The study excluded women and men who were under 18 years because legally, they are minors and could not give independent consent to participate in the study. Men and women who had their contraceptive needs met were excluded. In addition, snowball sampling was utilised to identify potential participants who could not be identified through purposive sampling. Subsequent theoretical sampling of healthcare workers and men was determined by the evolving theoretical framework. Final sample size was determined by theoretical saturation.

### Data collection

Data were collected between November 2017 and August 2018. Face to face individual semistructured in-depth interviews with women and men were conducted in local language at a place and time convenient to them. Most interviews were conducted in participant’s homes whilst few preferred to be interviewed at the health centre. Interviews with healthcare workers were conducted at their workplace in English as English is the official language of communication at the workplace. Interviews were directed by topic guides that contained general, open-ended questions. Topic guides were specifically developed for this study based on the existing literature. Example questions included ‘*what are your views and perceptions about family planning methods?* and *what are the barriers that constrain women from accessing family planning methods?* Probing was done to elicit further details from participants. With progressing theoretical sampling, analysis and emergence of categories, interview questions became focused. Non-participant observations of family planning clinic consultations were conducted at the three health centres. The aim was to uncover the behaviours and interactions between healthcare workers and women as they accessed family planning services. All interview data were audio-recorded, transcribed verbatim and translated into English by the first author. Back translation of English transcripts into original Chichewa language was done to validate accuracy of translated interviews.

### Data analysis

Data were analysed manually. This process was informed by the principles of constant comparative technique and a three-stage hierarchical coding process: open, axial and selective coding procedures.^12^ In keeping with grounded theory, data collection and analysis were done concurrently. Codes and categories were inductively developed. Transcripts were read line-by-line to get sense of their meaning whilst generating codes. With further theoretical sampling, similarities and differences were noted in the codes. These codes were clustered to create categories and their properties and characteristics. Using constant comparison technique, the categories were compared to generate more inclusive categories. The inclusive categories were able to explain the relationships between the categories. Memos were documented throughout the process of data collection and analysis to denote the emerging codes and categories. Rigour was enhanced through regular meetings with the co-authors. Transcripts were shared and reviewed by the team to enhance theoretical sensitivity. Self-reflective accounts were documented throughout the process of data collection and analysis to keep track of the researcher’s reflections, assumptions, beliefs and feelings on the data and participants. This was important as it helped to enhance credibility and rigour of the findings of this study, given that all researchers are midwives, and women with children, one of whom lives and works in Malawi.

### Ethical consideration

The study was approved by the University of Manchester Research Ethics Committee (2017-2013-3592 redacted for peer review) and the National Committee for Science and Technology in Malawi (P.10/17/219 redacted for peer review). Furthermore, permission was sought from the Lilongwe District Health Office, nurse managers of the health centres as well as individual participants. Participants were given written information sheets, in local language with details of the study. Those that were willing to participate in the study provided written consent. Participants’ identities were anonymised using pseudonyms of their choice and observational data were anonymised using a code. Only authors of this manuscript had access to the data.

## Results

### Characteristics of participants

**FIGURE 1:**
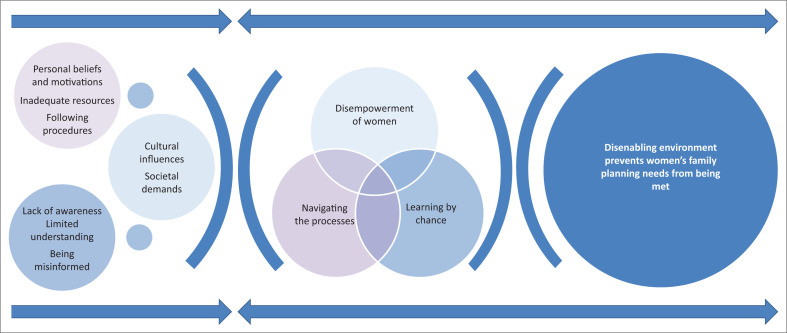
Disenabling environment prevents women’s family planning needs from being met.

A total of 38 interviews were conducted with 18 community women, 10 community men and 10 healthcare workers. Twenty women were approached to participate in the study; two declined without providing any reasons. None of the healthcare workers or men who were approached declined to participate in the study. Baseline characteristics of participants in this study have been summarised in Tables 1 and 2. The tables indicate that participants were of differing ages and employments. All community women and men were married, and they had an average of three children. The majority of participants were Christians. Although all community participants could mention a method of contraception, none of them were using any family planning method at the time of conducting this study. All healthcare workers were based in the maternal and child health department. This is the department that provides maternal and child health services such as family planning, cervical cancer screening, antenatal care, labour and birth services including postnatal care. Eight out of ten healthcare workers had undergone post qualification training in family planning provision. Provision of family planning methods was their core responsibility. In-depth interviews lasted between 30 minutes and 1 hour, whereas actual clinic appointment non-participant observations lasted between 5 min and 15 min. The core category: ‘disenabling environment prevents women’s family planning needs from being met’ was developed (Figure 1). The core category embodied three main subcategories: ‘navigating the processes’, ‘disempowerment of women’ and ‘learning by chance’.

**TABLE 1:** Baseline characteristics of study participants.

Variable	Women	Men
Mean age	28.7	31.6
Total number	18	10
**Occupation**		
Housewife	14	0
Small-scale business	3	2
Manual work	0	4
Clerical	0	4
Professional	1	0
**Marital status**		
Married	18	10
Single	0	0
**Current use of contraception**	0	0
**Mean number of live children**	**3**	**3**

**TABLE 2:** Baseline characteristics of healthcare providers.

Variable	Participants (*N* = 10)
**Gender**	
Male	2
Female	8
Mean age	33.3
**Qualification**	
Master’s degree	2
Bachelor’s degree	3
Diploma	4
Malawi school certificate	1
**Professional Cadre**	
Registered Nurse/Midwife	6
Enrolled Nurse Midwife	3
Health Surveillance Assistant	1
**Post qualification training in family planning provision**	
Yes	8
No	2

### The major categories

#### Navigating the processes

‘Navigating the processes’ in Figure 1 demonstrates the journey it takes for a woman to access family planning services at the health centre. Accessing family planning services was not a straightforward process. The process seemed to be influenced by several factors, which were clustered into three subcategories, namely *personal beliefs and motivations, following procedures* and *inadequate resources.*

Most women reported positive insights towards contraceptive use with a few reporting that they had never used any contraception. One participant expressed her confidence on the effectiveness of modern contraceptive methods as illustrated:

‘I can say that family planning methods are good because, as a woman you are not at risk of having an unwanted pregnancy if you are having unprotected sexual intercourse. There is nothing to fear because you are protected.’ (Esnat, female, 29 years, policewoman)

Similarly, some men discussed constructive perceptions towards family planning methods. They reported on the health benefits of using contraception, which motivated some couples to continue using contraception:

‘Family planning methods are good because a woman is healthy because she does not bear children frequently. As a family, we decide on the number of children to have.’ (Yohane, male, 40 years, manual worker)

However, many participants across the three groups explicitly alluded to the notion that the use of family planning methods was detrimental to the health of the woman. One healthcare worker stated her concerns about the effects of modern contraceptives:

‘Personally, as an individual, I don’t like family planning methods. I only tried Depo-Provera once and I bled a lot. So, from that time I never used any family planning method.’ (Grace, female, 33 years, registered nurse midwife)

Participants voiced their dissatisfaction with the care that they received at the family planning clinic. Women who reported to the clinic on an initial visit for contraception had to follow various procedures before accessing the services. This was done to assist healthcare workers to confirm eligibility of a woman to use a family planning method. As stated by one community woman, the procedures were restrictive for a woman to access contraception:

‘I was told to come to the clinic only when I had my period back. When I told her that I am not pregnant, she started shouting at me. They had to send me back after I was already on the couch.’ (Esther, female, 38 years, small scale business woman)

Confirming what Esther noted, one healthcare worker acknowledged this practice and how it negatively affects women:

‘We tell them to come to the clinic only when they are having menses without considering their concerns of distance and lacking transport money.’ (Fanny, female, 30 years, registered nurse midwife)

Access to family planning methods was only available after having a mandatory blood test. It was noted that the test was performed on all people seeking health services at the health centres:

‘We have the strategy of 90-90-90 strategy, 90% of the Malawian population should know their HIV [human immunodeficiency virus] status, 90% should start antiretroviral therapy [ART], and the 90% on ART should have a viral suppression, so in order to achieve that target, we need to be testing everyone who comes to the facility for HIV …’ (Max, male, 28 years, registered nurse midwife)

Given the importance of this test, one healthcare worker stated her concerns about the negative influence of the compulsory HIV (human immunodeficiency virus) test. She emphasised widespread uncertainties amongst the women in accessing family planning services:

‘I also heard from some women that their friends are not coming for family planning methods because of fear of being tested for HIV.’ (Lusungu, female, 40 years, registered nurse midwife)

Access to permanent and long-term family planning methods seemed to be performed under certain limitations. One community woman stated her frustrations when healthcare workers challenged her prospects to undergo tubal ligation:

‘Soon after delivering this child, I wanted a permanent method to be closed, and for my tubes to be cut. I signed the form to go for the closing but the nurse on duty refused me to go for tube cutting since I am still young.’ (Linje, female, 36 years, small scale business lady)

Although all family planning methods were reported to be available at each health centre, it was reported that there was lack of a skilled workforce to provide long-term family planning methods. One healthcare worker reflected on how this affected her family planning service provision:

‘Like myself and other four nurse midwives have not been trained in provision of long-term family planning methods. In this case she [woman] has no choice so we send the women back home without a service.’ (Linda, female, 28 years, enrolled nurse midwife)

Given the importance of contraceptive use in averting unplanned pregnancies, its scarcity elevated a woman’s risk of having unintended pregnancies. Another man was concerned that his wife would not have access to her contraception of choice any sooner. He stated:

‘My wife is not using any method because the injection is out of stock at the health centre. She had one injection last year, up to now she is not on any method.’ (Levison, male, 27 years, manual worker)

#### Disempowerment of women

As illustrated in Figure 1, this category embraced two subcategories: societal demands and cultural influences. The category provides an explanation of women’s and men’s thoughts regarding the influence of religious beliefs, culture and society on women’s contraceptive decisions. Most of women’s thoughts were grounded in their explanations of lacking empowerment from their spouses and significant others in society to use contraceptive methods.

Societal demands demonstrate the perceptions that people in society shared collectively as being a gender norm in terms of assigning responsibilities related to contraceptive use and childbearing. Men’s perceptions on their role in family planning and childbearing remained unclear. One man questioned the relevance of men to be involved in family planning issues:

‘Aaaa the thing is that a man does not bear children, it is only a woman who bears a child so, why should a man use contraception?’ (Lameki, male, 38 years, small scale business man)

Having many children was perceived to be in alignment with societal expectations of childbearing. As a result, some men considered childbearing to be rewarding. Accordingly, couples feared the ridicule, which was a commonplace in society for couples who have challenges to conceive:

‘What happens is like we are in a competition to have as many children … They would also tease a man to allow other men to make the wife pregnant if the woman is failing to conceive.’ (Mike, male, 25 years, manual worker)

The quotation from Mike illustrates that men perceive that a woman’s role is to bear many children in society.

At the other end of the continuum, women spoke of the negative attitudes of their partners towards contraceptive use. For instance, Elas discussed how prohibitive her husband was towards contraceptive use:

‘I am planning that I have the injectable contraceptive so that he does not know that I am on contraception. I will do this because he does not allow me to use any contraceptives …’ (Elas, female, 18 years, housewife)

Consistent practices of disapproving contraceptive use were also recognised by significant others in society. These significant others had substantial decision-making power over contraceptive use and family size as illustrated in this quote:

‘The other factor influencing women is the influence of relatives of the husband. They tell the woman that they desire a good number of children. So, as a woman, we do not have power to say no to this.’ (Margaret, female, 30 years, housewife)

Cultural influences referred to the cultural beliefs, religious beliefs and other patterns of behaviours that were shared amongst the people in society who either promoted or hindered contraceptive use. Most participants felt that cultural and religious beliefs informed their contraceptive practices.

Both men and women acknowledged the religious teaching that restricts the use of contraception, thus rendering women to be cautious to use contraception:

‘What I know is that the Bible says that we should multiply like sand meaning that it is forbidden for one to use these methods, it is against the Bible teachings …’ (Rachel, female, 26 years, housewife)

In contrast, other religious doctrines recognised the need for families to use family planning methods to avert unwanted pregnancies. This permitted some of its followers to be motivated to use contraception as stated:

‘I belong to the Muslim faith. There are no restrictions on using family planning contraceptives. Actually, women are encouraged to use family planning methods.’ (Patricia, female, 35 years, housewife)

Some participants reported of using unconventional methods to prevent pregnancies. For instance, one man expressed his confidence in using traditional medicine as illustrated in the following excerpt:

‘My wife and I have seven children together and she has been using this traditional method with all these children; and I am yet to have more children.’ (Yankho, male, 36 years, clerk)

Signifying the efficacy of traditional medicine to prevent unwanted pregnancies, one woman testified the usefulness of traditional medicine in offering long-term protection from pregnancy:

‘I have been using traditional medicine which I was getting from the traditional healer. He prepares a medicated rope from the bulk of the tree. If the rope contains 3 knots, it means I will be protected from pregnancy for three years.’ (Doris, female, 35 years, housewife)

#### Misinformation and lack of understanding

This category describes the knowledge gap that exists regarding contraceptives that was revealed by participants. Although both men and women could name at least one family planning method, they showed lack of understanding of how the contraceptives work:

‘Oky there is the injection, pills, Norplant. I cannot mention in detail how these methods work because I have never used them before.’ (Doris, female, 35 years, housewife)

Equally, some men cited lacking awareness of family planning methods including their benefits. However, some mentioned vasectomy as a method of permanent contraception for men. One man had scanty knowledge of how vasectomy is performed, thus increasing his fears to go for the procedure:

‘What I know is that they cut a certain part of a man’s private parts. A lot of men including me I do not know much about it.’ (Biliati, male, 26 years, clerk)

During family planning consultations, most healthcare workers would not provide one-to-one counselling regarding the family planning methods (Observational field note): Chikumbutso told the nurse provider that she had never heard of such contraception (implant). However, the nurse inserted the implant without informing Chikumbutso what the contraception was all about and what to expect.

Some participants’ decisions to use or not to use contraception were grounded from distorted messages regarding what contraceptives would do to a person’s body. In general, most participants indicated lack of correct information in relation to what contraceptive methods would and would not do to the body as illustrated:

‘The other bad thing that I have heard is that when they want to insert a Norplant, they make a big cut so that they insert it.’ (Elina, female, housewife)

One participant was scared to hear that a woman who had used contraception for a long time would develop cervical cancer, which could be transmitted to her spouse:

‘I have heard that a woman who is using contraception is at risk of suffering from cervical cancer. Eventually, this woman will transmit the cancer to her husband.’ (Mike, male, 25 years, manual worker)

## Discussion of findings

This study advances the understanding of the experiences of women accessing family planning services in Malawi from multiple lenses. Participants cited that they would only be initiated on contraception when they reported to the clinic during their menses to ensure that the woman was not pregnant. If they reported to the clinic when they were not having their menses, they were asked to go to a private clinic to have a pregnancy test. However, many women could not afford to have the pregnancy test at a private clinic because of financial challenges. This finding is supported by a study conducted in Senegal, which reveals that healthcare workers are hesitant to initiate a woman on contraception if she is not in menses.^14^ They argue that healthcare workers fear that contraception would harm an unrecognised pregnancy especially if the woman opts to have an IUCD.^14^ However, this practice contradicts guidelines provided by the World Health Organization (WHO).^15^ The WHO suggests that healthcare workers can initiate a woman on contraception when the woman is reasonably sure that she is not pregnant in reference to the pregnancy checklist when providing family planning services.^15^ However, in our study, none of the healthcare workers were observed referring to the pregnancy checklist when providing family planning services. Therefore, our study suggests that healthcare workers ought to utilise the pregnancy checklist as proposed by WHO to rule out pregnancy during a family planning consultation session before initiating a woman on contraception. This would help reduce unnecessary costs of having a pregnancy test from a private clinic by women. Moreover, the use of a pregnancy checklist would reduce missed opportunities for contraceptive use as women would not be sent back home to await menses.

Our study has established that provision of family planning services at the three health centres was compromised by contraceptive supply challenges. The most liked contraceptive method amongst the women, Depo-Provera, was frequently out of stock. Consequently, many women were frustrated and were left without contraception. Various studies have highlighted that limited availability of contraceptive methods negatively affects provision of family planning services.^16,17,18^ Interventions that provide a constant supply of pregnancy test-kits and contraceptives may help guarantee timely provision of family planning services.

A unique finding from this study revealed that women could only access family planning services after having a mandatory HIV test for early detection of HIV and initiation of ART. Early initiation of ART is claimed to improve the overall health outcomes and longer life expectancies amongst people.^19^ However, imposing an HIV test on women may create a missed opportunity for contraceptive use. Healthcare workers indicated that HIV testing was mandatory for a woman to access family planning services. However, the guidelines stipulate that HIV testing ought to be performed on a voluntary basis after careful counselling and when a person consents to carry out the test.^20^ The clinical implication for this mandatory test is that some women who are not ready to have the HIV test performed may shy away from the alleged mandatory HIV testing. Consequently, women may not go for contraception for fear of being tested for HIV. This would negatively influence the achievement of the 90-90-90 strategic goals. However, the impact of mandatory HIV testing on uptake of family planning services in Malawi remains unexplored. Nevertheless, implementation of the 90-90-90 strategy as specified in the National Strategic Plan for HIV and AIDS^20^ would help ensure uniformity on the practices of healthcare workers. This would enable women to decide to consent to have an HIV test or not. Also, the goals of the strategy might be achieved and increase contraceptive usage.

Some healthcare workers acknowledged lacking training and expertise to offer implants and IUCDs to women. As a result, women who opted to have these long-term contraceptives were sent home without a service. Lack of training created an unnecessary barrier for contraceptive use. These findings mirror those by other researchers who found that healthcare workers lack skills to provide long-acting implants and IUCDs.^21,22^ Likewise, in Nepal some nurses are not confident to provide family planning services despite receiving training.^23^ These findings highlight the need for the Ministry of Health to invest in building the capacity of healthcare workers to enable them to provide all contraceptive methods available at health centre level. Training healthcare workers would equip them with knowledge and skills and increase their expertise and enthusiasm to provide various family planning methods.

Disempowerment of women had a fundamental impact on the realisation of women’s family planning needs. Men were the primary decision makers in a marital relationship. Consequently, couple support for contraception was inadequate as most men disapproved contraceptive use. Accordingly, women assumed a subordinate role by respecting their husband’s opinions and became cautious to use contraception. This finding is consistent with an existing study, which highlights the importance of having more children to raise the status of an individual.^24^ Similarly, in Democratic Republic of Congo (DRC) and Ethiopia, societies recognise the need for families to have many children (as low as 4, as high as 8–10 in DRC) who are a free source of labour.^25,26^

This study has highlighted the role of traditional medicine and unconventional methods in averting unwanted pregnancies. This finding mirrors those of studies conducted elsewhere, which affirm that because of fear of side-effects of modern contraception, some women used concoctions or ampicillin immediately after sexual intercourse to avert unwanted pregnancies.^27^ However, the efficacy of using traditional medicine and antibiotics to avert pregnancies has not been proven and could cause adverse health effects and drug resistance.

## Recommendations

Our study suggests the need for interventions by the Ministry of Health to address health system constraints to ensure sustained use of contraception. Because of unavailability of pregnancy test-kits, the study proposes that healthcare workers should utilise the pregnancy checklist as proposed by the WHO to rule out pregnancy before initiating a woman on contraception. This would help reduce missed opportunities for contraception.

Likewise, investing in capacity building of healthcare workers is recommended. In-service training would enable healthcare workers to provide all contraceptive methods available at health centre level, hence increase women’s choices of contraception.

There is a need for healthcare workers to have further training on how to implement the 90-90-90 HIV strategy to ensure uniformity in its implementation. This will also help minimise missed opportunities for contraception for women who are not ready to undergo mandatory HIV testing.

Our research suggests the need to elevate the status of women in society to ensure gender equity in making decisions at all levels. This could be done by developing and enforcing laws that would promote gender equity so that a woman’s voice to use contraception could be heard. Strategies to strengthen health education and counselling services at community level are suggested to promote awareness of contraception.

Since women had to undergo mandatory HIV testing before accessing contraception, our study recommends that further research be undertaken to explore the impact of mandatory HIV testing on uptake of family planning services in Malawi.

## Limitations

This grounded theory study had some limitations. The authors were aware of the impact of personal beliefs, assumptions and experiences on the research process which could influence how data were interpreted. Efforts were made to minimise this by maintaining reflexive accounts of beliefs and experiences throughout the research process. Accordingly, the authors kept a record of responses to data in the form of memos and field notes.

Findings from this study may not be transferable to all health facilities in Malawi because the study was limited to participants who patronise public health centres. Participants from private health facilities were not considered in this study. Furthermore, non-inclusion of unmarried clients and those with a met need for contraception might have led to the omission of positive perspectives that positively influenced gaining access to contraceptives, which could guide further interventions in the study. Notwithstanding these limitations, findings from this study have important implications for family planning practice, education, policy and research in Malawi.

## Conclusion

The findings of this study indicate that accessing family planning services in Malawi is not a straightforward process. Women face challenges related to limited resources as well as procedures set by the healthcare system. Drawing on the ‘disenabling environment’ framework in Figure 1, provision of family planning services needs to be tailored towards an ‘enabling environment’ which would promote contraceptive use in Malawi. There is need for increased prioritisation of family planning services by the Ministry of Health; so that contraceptive commodities are consistently available in all health centres. This would enhance timely provision of family planning services for women. Nevertheless, capacity building for family planning providers is required so that they acquire skills to provide a range of family planning methods at the health centres. Healthcare workers need further in-service training on the implementation of the 90-90-90 HIV strategy to endure uniformity in their practice.
